# A pilot open-label feasibility trial examining an adjunctive mindfulness intervention for adolescents with obesity

**DOI:** 10.1186/s40814-020-00621-1

**Published:** 2020-06-06

**Authors:** Elizabeth W. Cotter, Sarah E. Hornack, Jenny P. Fotang, Elizabeth Pettit, Nazrat M. Mirza

**Affiliations:** 1 Department of Health Studies, American University, 4400 Massachusetts Ave NW, Washington DC, 20016 USA; 2 Division of Psychology and Behavioral Health, Children’s National Health System, 111 Michigan Avenue NW, Washington DC, 20010 USA; 3 Department of Psychology, American University, 4400 Massachusetts Ave NW, Washington DC, 20016 USA; 4 George Washington University School of Medicine and Health Sciences, 2300 Eye Street, NW, Washington DC, 20037 USA; 5 IDEAL Pediatric Weight Management Clinic, Children’s National Health System, 111 Michigan Avenue NW, Washington DC, 20010 USA

**Keywords:** Obesity, Adolescence, Mindfulness

## Abstract

**Background:**

Obesity in adolescence is predictive of obesity in adulthood and risk for chronic disease. Traditional behavioral approaches to addressing obesity in adolescence rarely yield meaningful changes in body mass index (BMI), suggesting that adjunctive treatments are necessary. Herein, we describe a study examining whether it is feasible to integrate a brief mindfulness intervention with the usual recommended care for adolescent obesity in a pediatric weight management clinic.

**Methods:**

We conducted a single arm open-label trial with 11 adolescent patients with obesity. Participants received the recommended standard of medical management of obesity (usual care) plus a six-week mindfulness intervention. To assess our primary aim of feasibility, we examined recruitment, retention, and satisfaction rates. Participants also completed measures of mindfulness, emotion regulation, disordered eating, quality of life, and executive functioning, and had their BMI and blood pressure measured.

**Results:**

We recruited 11 adolescents to participate in the intervention, with 8 (73%) completing the entire program. Attendance rates (85%) and satisfaction rates (100%) were promising for a larger trial. While preliminary analyses of changes in health outcomes should be examined with caution, effect sizes ranged from small to large with some promising trends in eating behaviors.

**Discussion:**

It might be feasible to augment existing behavioral interventions for adolescents with obesity with brief mindfulness; however, some adaptations are needed to enhance recruitment and retention. The lessons learned in this feasibility study can inform an adequately powered efficacy trial.

**Trial registration:**

This research is registered on ClinicalTrials.gov (NCT03874377).

## Introduction

Obesity in adolescence is directly related to risk for cardiovascular disease and type 2 diabetes mellitus [1, 2]. Traditional behavioral approaches to treating obesity assume that risk can be reduced through adolescents’ adoption of healthy lifestyle behaviors [3]. At present, standard of care lifestyle management interventions for adolescents with obesity do not reliably yield significant changes in weight loss [4], suggesting that adjunctive treatment components are necessary [5]. This paper describes a pilot open-label trial to examine the feasibility of a brief, adjunctive mindfulness intervention tailored to the needs of adolescents with obesity.

Adolescence is a time of dramatic change as youth go through puberty and experience a number of neurobiological changes that influence their behavior and decision-making [6]. Adolescents face stressors unique to their age group, such as initiating romantic relationships and gaining autonomy from caregivers, that can lead to maladaptive coping strategies if not addressed properly [7]. Mindfulness appears to be a particularly promising strategy in helping adolescents navigate these challenges and cope with them affectively. Mindfulness refers to the experience of paying attention to the present moment in a nonjudgmental manner [8]. Existing literature regarding the receptiveness of adolescents to mindfulness-based interventions (MBIs) indicates that adolescents generally are accepting of mindfulness practice [9]. When analyzed for efficacy, MBIs designed for adolescent populations have been shown to improve self-regulation [10], exercise levels [11], depressive symptoms [12], and resilience [13]. Conducting MBIs with adolescents also has some potential drawbacks, including poor attendance when programming is optional [14], participants’ difficulty adhering to MBI regimens [15], and a lack of standard adaptations for MBIs in adolescent populations [16].

Mindfulness-based strategies have been successfully incorporated into weight loss and weight management interventions with adults [17, 18], grounded in the understanding that mindfulness training enhances emotion regulation and one’s awareness of decision-making processes and internal experiences [19]. Emotion regulation difficulties in adolescents are associated with increased emotional eating and binge eating [20, 21]. Longitudinal research suggests that emotional eating influences risk for adiposity and metabolic disorders as adolescents move into adulthood [22–24]. Adolescents with higher impulsivity, or the tendency to act rashly when experiencing distressing emotions, are most likely to engage in emotional eating and binge eating [25]. Previous research indicates that impulsivity and emotion regulation, key drivers of disordered eating, are not necessarily stable traits and can be modified through behavioral interventions [26, 27]. Mindfulness is posited to reduce impulsivity and improve emotion regulation skills by allowing individuals to become more comfortable with negative emotions without hastily reacting to them [28]. Mindfulness has also been associated with changes in brain regions involved in emotion regulation and inhibition that are relevant to weight management [29]. MBIs have shown particular promise in improving obesity-related eating behaviors in adults—including binge eating and emotional eating—with effect sizes ranging from medium to large [18, 30].

At present, there is a scarcity of studies examining the effects of MBIs in adolescents with obesity (defined as a body mass index > 30 kg/m^2^), and it is unclear if such interventions are feasible for this population in tandem with standard medical management of obesity. Further, although research suggests that mindfulness can improve insulin resistance in adolescents with obesity [31], it is unknown whether mindfulness can influence other comorbidities in this population, including markers of cardiovascular disease (CVD) risk. Kumar et al. [32] conducted four, 90-min family-based mindful eating sessions with adolescents with obesity and their parents; however, while this program yielded promising retention and attendance rates, it did not yield significant changes in blood glucose, BMI, or total cholesterol. In contrast, a mindful eating intervention for Latina girls yielded reductions in BMI, but retention rates were only 57% [33]. Therefore, in addition to examining the feasibility of this intervention in a hospital weight management clinic setting, we will also examine the preliminary effects of the MBI on health gains including quality of life and CVD biomarkers associated with obesity in order to establish the potential for change.

The proposed study had two specific aims: (1) to examine the feasibility of conducting an open-label trial of the mindfulness intervention in a sample of 15 adolescents with obesity, including recruitment, retention, and satisfaction rates; and (2) to establish the potential for change through the measurement of clinical outcomes including BMI, emotion regulation, eating- and weight-related behaviors, quality of life, impulsivity, and blood pressure at baseline and post-intervention.

## Study design and methods

### Study setting

This study took place at a multidisciplinary, research-oriented weight management clinic located within a large children’s hospital in the Mid-Atlantic during February through August of 2019. This clinic accepts patients with obesity aged 6 months to 21 years. Approximately 100 new patients are seen annually. Approximately 39% of the patients seen are Black or African Americans, 28% are Hispanic American, and 20% are White Americans. Sixty-five percent of the patients have Medicaid, and 34% are private or commercially insured. The clinic follows the recommended standard of medical management of overweight and obesity. The clinic team conducts a comprehensive evaluation to assess dietary and activity behavior change needs of each patient and family, as well as obesity-associated comorbidities. In addition to management of comorbidities, goals for improved physical activity and dietary behaviors are set with the patient and family at each visit.

### Inclusion and exclusion

Adolescents were eligible if they were between the ages of 12–17, a current patient of the affiliated obesity clinic, and had a BMI greater than 30 kg/m^2^. Adolescents were ineligible if they had a known genetic cause of obesity, had been diagnosed with a severe intellectual or learning disability, had been diagnosed with an autism spectrum disorder or current psychosis, or were currently in psychotherapy. These exclusion criteria were selected because they might influence the degree to which an adolescent responds to behavioral weight loss and/or their ability to actively take part in a standard mindfulness intervention.

### Recruitment

Medical providers and other clinic staff were informed about the study and referred interested patients’ parents/caregivers to the research team. Interested patients were screened for eligibility, and eligible patients were consented in the presence of a parent/caregiver and scheduled to complete a baseline assessment.

### Participant assessment schedule and measures

Participants completed a series of assessments within a private clinic room at the obesity clinic. These measures were completed at baseline and post-intervention, unless otherwise stated.

#### Feasibility outcomes

##### Assessing recruitment feasibility

Recruitment feasibility was examined through research staffs’ detailed tracking of recruitment processes (e.g., referrals from physicians, patients approached in the waiting room), the number of interested patients, and the number of patients eligible after the initial screening. We documented all cases of ineligibility and the reason for disqualification.

##### Assessing retention feasibility

Retention feasibility was tracked via attendance; research staff took attendance during each assessment period and intervention session. Any enrolled participants who dropped out (defined as participants who either explicitly stated that they would like to leave the program or who missed two consecutive sessions in a row without contacting the research team) were contacted by phone to inquire about their reasons for ending the program, as a method of assessing any barriers to intervention completion.

##### Assessing participant satisfaction

We assessed participants’ satisfaction with the mindfulness intervention by having them complete a satisfaction survey at the end of the intervention. This survey evaluated the following: (1) reactions to the topics discussed and skills reviewed, (2) comfort with facilitators; (3) opinions of the materials used, and (4) overall satisfaction with the intervention at that point in time. Ten of these items were scored on a Likert-scale of 1 (strongly disagree) to 5 (strongly agree) (e.g., “Participating in this program helped me to better manage my eating habits”) and eight of these items invited open-ended responses (e.g., What were the challenges of participating in this study?). Participants were also asked to report their favorite and least favorite components at the end of each session. This feedback will inform potential adaptations made to the intervention prior to a larger randomized controlled trial (RCT).

#### Participant self-report measures

##### Demographic questionnaire

Demographic questionnaire assesses age, gender, and race/ethnicity. This measure was only completed at baseline assessment.

##### Mindful attention awareness scale-adolescent

The Mindful Attention Awareness Scale-Adolescent (MAAS-A) is a 15-item measure of dispositional mindfulness. Participants rated how frequently they experience episodes of mindless behavior (e.g., “I find myself doing things without paying attention.”) on a scale of 1 (almost always) to 6 (almost never). This measure has established psychometric properties in diverse adolescent samples [34–36].

##### Eating disorders examination-questionnaire

Participants reported instances of overeating, loss of control eating, and binge eating over the last 4 weeks via 3 items of the Eating Disorders Examination-Questionnaire (EDEQ) [37]. This measure was selected to examine potential changes from baseline to post-assessment in eating behaviors that are associated with emotion regulation and risk for obesity that might be influenced by mindfulness training [22–24]. The EDEQ yields reliable and valid scores [38]. Adolescent responses to the EDEQ correspond strongly with clinical interviews assessing disordered eating [39].

##### Difficulties in emotion regulation scale–short form

The Difficulties in Emotion Regulation Scale–Short Form (DERS-SF) is an 18-item, widely used self-report measure of emotion regulation problems that has been validated in adolescent samples [40]. This measure was selected to examine potential changes from baseline to post-assessment in emotion regulation, which is theorized to be a key driver of disordered eating (e.g., binge eating, overeating) in adolescents with obesity [41].

##### Youth quality of life instrument–short form

The 15-item Youth Quality of Life Instrument–Short Form (YQOL-SF) measures generic quality of life in youth with and without chronic conditions, ages 11–18 years. It has established psychometric properties in adolescent samples [42, 43].

#### Executive function measure

##### Go/no-go task

The Go/No-Go Task examines inhibitory control via a computerized program. This measure was selected to examine potential changes from baseline to post-assessment in impulse control, which is associated with binge eating [25] and obesity [44], to provide evidence of the cognitive mechanisms through which MBIs might improve eating behaviors. Participants are instructed to press a button (or “Go”) when a certain image is shown on the screen (i.e., an image of food). They are instructed not to respond (or “No-Go”) when another image is shown on the screen (i.e., an image of a toy) [45]. The entire task is approximately 15 min, and each image is shown on the screen for approximately 500 ms. Poor impulse control is evident by more failures to inhibit responses in the No-Go condition (e.g., a false alarm). Omission occurs when a participant fails to respond to a Go stimuli. Reaction time is the processing speed for correct Go trials. This task demonstrates reasonable reliability and validity in adolescent samples [46, 47].

#### Anthropometric and CVD biomarker measures

##### Anthropometrics

Height and weight were assessed in order to calculate BMI. Height was measured to the nearest 1/8 in. using a wall-mounted stadiometer. Weight was measured in indoor clothing, without shoes, to the nearest 0.1 lb using a calibrated digital scale.

##### Blood pressure measurement

Blood pressure was measured using an automatic upper arm cuff while the participant was seated. Participants were instructed to sit quietly, with both feet uncrossed on the floor, and their arm in a still position, to increase accuracy of each measurement.

### Intervention

The mindfulness intervention consisted of 6 weekly sessions, blending material from the evidence-based Learning to BREATHE [48] and Mindfulness-Based Eating Awareness Training [19] manualized interventions. Participants met individually with a therapist for each 60-min session. Sessions focused on the following: experiential mindfulness exercises, such as mindful eating, loving kindness practices, breath awareness, and mindful movement; hunger and satiety awareness; improving responses to emotions; practicing acceptance and being non-judgmental; and tolerating negative feelings and sensations, including those related to hunger and cravings. Participants were assigned brief homework exercises (approximately 10 min) daily in between appointments. Sessions were offered via telemedicine to improve attendance.

### Adverse events and criteria for discontinuation

Expected risks to participants in this study were generally mild. There was the unlikely but possible risk that participants could experience negative emotions during the practice of mindfulness. Participants were informed about this risk during the consent process and encouraged to inform a member of the research team if they had a strong negative reaction. Mindfulness facilitators were trained to provide adaptations to the mindfulness exercises in order to reduce the intensity of these experiences and ground the participant in the present moment. If an individual continued to experience intense negative emotions that interfered with their ability to participate in the program despite these adaptations, they would have been withdrawn from the study and provided with appropriate referrals. The investigators and research staff met regularly to discuss participants’ reactions to the assessments and intervention and any study withdrawals. No adverse events were reported.

### Facilitators and training

The mindfulness components of the intervention were facilitated by two interventionists with established mindfulness practices, including one graduate-level clinical psychology student and one first-year medical student. Each facilitator received extensive supervision from the first and second authors (both licensed clinical psychologists) via weekly meetings. Participants met with the same facilitator throughout the course of the intervention. The usual care components were facilitated by medical providers within the obesity clinic.

### Adequacy of sample size

Our study’s primary aim was to examine feasibility and refine aspects of the research approach; therefore, a formal sample size calculation was not warranted [49]. Given budgetary and logistical constraints, our aim was to recruit 15 adolescents over a 6-month period through a single clinic site, which is sufficient to provide useful information about the feasibility of the protocol [50].

### Data analysis

IBM SPSS Statistics version 24 was used to complete all quantitative data analyses. Frequencies were used to examine feasibility, including recruitment and eligibility rates, rates of attendance at each session and assessment appointment, and satisfaction with the intervention. Paired *t* tests were conducted to examine pre-post differences in the health outcomes of interest, and Cohen’s *d* effect sizes and confidence intervals were calculated to examine the effect size of any changes in BMI, mindfulness, emotion regulation, eating behaviors, quality of life, impulsivity, and blood pressure [51, 52]. For participants who did not complete post-assessment (*n* = 3), we used a last observation carried forward imputation.

### Data security

All participant data was kept in a secure locked location in the PI’s research lab. Participant data was also stored electronically on an encrypted data “cloud” that was accessible only from university servers, which is firewalled and password protected to guard against data loss or theft and to avoid any potential breach in subjects’ privacy and confidentiality. Participant names were replaced with identification numbers to maintain confidentiality. Only study staff had access to identifiable information, and all of them were required to complete the Collaborative Investigator Training Initiative (CITI) course in human subjects’ protection.

## Results

### Participants

**Fig. 1:**
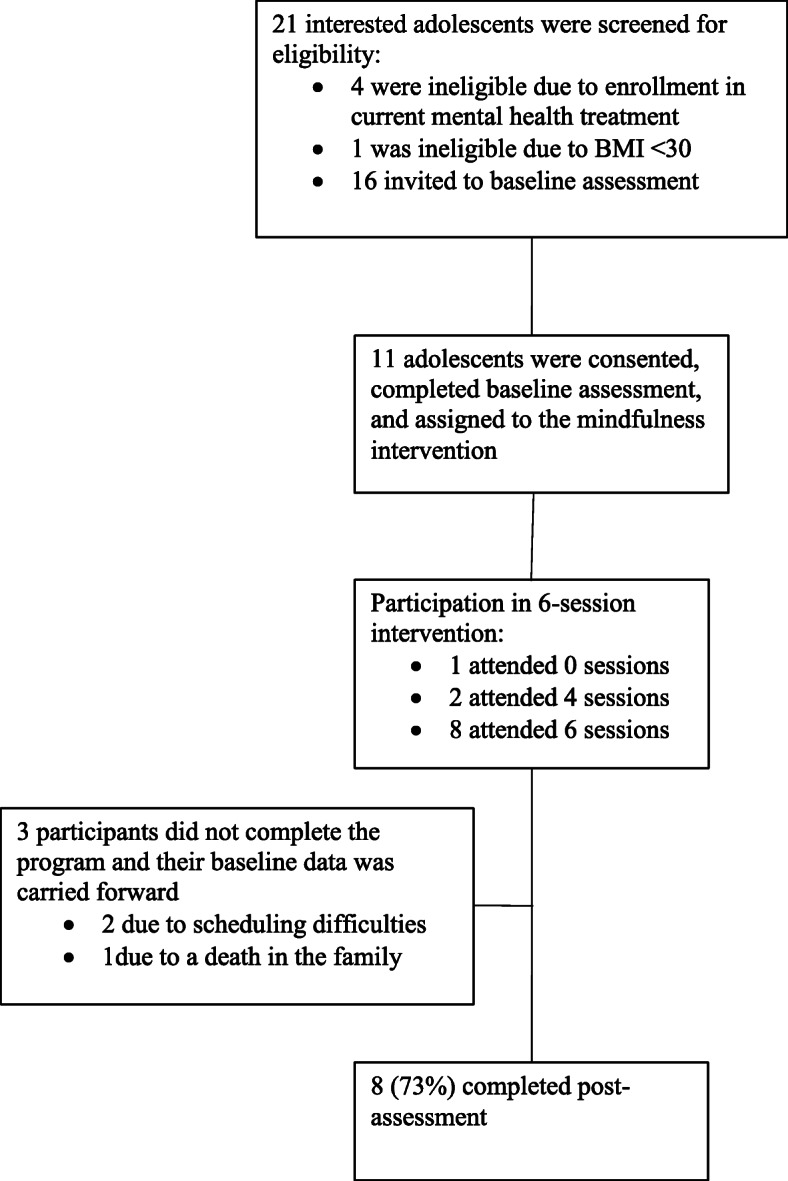
Study participant flow chart

Figure 1 depicts the flow of study participation. Twenty-one adolescent patients of the obesity clinic were screened for eligibility. Five of these patients were ineligible for the study because they were currently in mental health counseling (*n* = 4) or had a BMI under 30 (*n* = 1). Sixteen (76%) screened adolescents were eligible and 11 enrolled in the study (73% female; 64% Black/African American, 18% Hispanic/Latino, 18% White; *M*_age_ = 14.36, SD = 1.90 years; *M*_BMI_ = 35.70, SD = 5.28; BMI range 31.37 to 50.30). Eight of the 11 enrolled participants (73%) completed post-assessment.

### Feasibility

Table 1 presents recruitment, retention, attendance, and satisfaction rates. Recruitment was slower than expected, and we did not reach our goal of 15 adolescents (final enrollment was 11 adolescents). Retention rates were slightly below expectations (73% versus an expected 80%), while attendance and satisfaction rates were promising (85% and 100%, respectively). Regarding preferred modality of intervention delivery, 8 participants opted to complete the intervention via telemedicine, 2 participants completed the sessions using a mix of in-person and telemedicine appointments, and 1 participant completed the intervention completely face-to-face. Participants were asked to rate their satisfaction with various aspects of the program on a scale of 1 to 5, with higher scores representing greater satisfaction. Participants responded most favorably to items assessing comfort with program staff (*M* = 5, SD = 0), improved ability to explain mindfulness to others (*M* = 4.50, SD = .76), and improved ability to be mindful on one’s own (*M* = 4.50, SD = .53). Participants responded least favorably to an item assessing their use of mindfulness in their daily life (*M* = 3.88, SD = .64). Table 2 presents participant open-ended responses regarding self-reported benefits of participation, challenges to participation, and changes noticed in oneself post-participation. Additional file 1 includes all participant responses to open-ended satisfaction items.

**Table 1:** Feasibility results

Measure	Target	Observed	Description
Recruitment	15	11	Twenty-one adolescent patients were screened for eligibility. Sixteen (76%) screened adolescents were eligible and 11 enrolled in the study over a 6-month period.
Retention	80%	73%	Three enrolled participants dropped out of the program, due to scheduling difficulties (*n* = 2) or for family reasons (*n* = 1).
Attendance	85%	85%	On average, participants completed 85% of the 6 intervention sessions. Fifty-four percent (*n* = 6) attended all of the required sessions.
Satisfaction	90%	100%	Participants were asked to rate their satisfaction with various facets of the program on a scale of 1–5. All of the participants who completed the satisfaction scale averaged a score of 4 or higher across these items, indicating they were satisfied with the program.

**Table 2:** Sample of Participant Quotes Post-intervention

Area	Sample Quotes
Benefits of the program	“Helped control eating and portions” “Learning when I’m actually hungry and when I’m stress eating” “Learning about mindfulness and sharing it with others” “Help[ed] manage weight loss and emotions” “Losing weight and watching what I eat”
Challenges of the program	“Coming every Tuesday” “Wishing good to people who have been bad to me” “Remembering homework” “Finding a good time [for appointments]” “Freeing up time each week”
Changes noticed in self	“I’ve become more peaceful and able to control my emotions” “Being able to calm down easier” “Thinking about strategies before healthy choices” “Falling asleep easier” “Mindfulness and meditation in my daily routine”

### Examination of health-related outcomes

Paired samples *t* tests were used to examine potential differences from baseline to post in BMI, mindfulness, emotion regulation, eating behaviors, quality of life, impulsivity, and blood pressure with the understanding that we were severely underpowered to detect significant differences. Table 3 presents each outcome, the means at baseline and post-assessment, the effect size, confidence intervals for the mean change, and the *p* value. The largest changes observed according to Cohen’s *d* calculations were in overeating (Cohen’s *d* = − .69, large effect size), and Go/No-Go Reaction Time (Cohen’s *d* = .78, large effect size). The only change that was significant was Go/No-Go reaction time (*p* = .05). The decrease in overeating was approaching significance (*p* = .07).

**Table 3:** Baseline and post means, effect sizes, and confidence intervals of health outcomes of interest

Variable	Baseline mean (SD)	Post mean (SD)	Difference CI 95% (lower, upper)	Cohen’s *d*	*d* CI 95% (lower, upper)	*p* value
BMI	35.70 (5.28)	32.79 (9.79)	− 3.38, 9.20	− 0.31	− 1.15, 0.53	.327
Blood pressure-systolic	116.00 (6.91)	117.22 (5.45)	− 4.48, 2.03	0.29	− 0.55, 1.13	.412
Blood pressure-diastolic	67.89 (9.37)	71.78 (9.99)	− 10.31, 2.54	0.47	− 0.38, 1.32	.200
Mindfulness	4.20 (0.75)	4.40 (0.95)	− 0.73, 0.32	0.29	− 0.55, 1.13	.402
Emotional regulation	2.49 (0.97)	2.45 (1.03)	0.10, 0.17	− 0.22	− 1.06, 0.62	.551
Over eating	8.33 (10.71)	6.50 (11.26)	− 0.22 , 3.89	− 0.69	− 1.55, 0.17	.073
Loss of control eating	2.95 (3.56)	2.25 (3.34)	− 0.53 , 1.93	− 0.41	− 1.25, 0.43	.230
Binge eating	5.70 (13.55)	1.95 (3.67)	− 3.46, 10.96	− 0.37	− 1.21, 0.47	.269
Quality of life	76.18 (14.09)	77.82 (13.47)	− 6.96, 3.69	0.21	− 0.63, 1.05	.509
Go/No Go false alarm rate	.45 (.22)	.39 (.19)	− 0.04, 0.17	− 0.41	− 1.25, 0.43	.202
Go/No Go omission rate	.04 (.06)	.04 (.06)	− 0.01, 0.003	0.32	− 0.52, 1.16	.315
Go/No Go reaction time (ms)	500.92 (69.11)	535.79 (70.94)	− 64.76, − 4.99	0.78	− 0.09, 1.65	.026

*Note:* Mean difference was calculated by subtracting post mean scores from baseline scores

## Discussion

Traditional behavioral weight management programs have limited efficacy in treating obesity in adolescence [4, 5]. MBIs might enhance the efficacy of existing behavioral interventions by enhancing adolescents’ cognitive functioning and emotion regulation processes [29], which in turn could improve eating and weight-related behaviors [17]. Our study examined whether such an intervention is feasible for adolescent patients with obesity, in preparation for a potentially larger efficacy trial. We also examined preliminary changes in impulsivity in adolescents (as measured through the Go/No-Go task), thus providing potential evidence of the cognitive mechanisms through which MBIs might improve eating behaviors. An additional innovation of our study was the focus on cardiovascular outcomes, although an adequately powered RCT is needed in order to understand whether MBIs might improve risk reduction for cardiovascular disease in adolescents with obesity regardless of weight reduction.

Our primary aim was to determine the feasibility of implementing this intervention in a pediatric outpatient weight management clinic. Although prior research with adolescents suggests that recruitment is generally feasible for adjunctive interventions in weight loss clinic settings [53], and for MBIs specifically [31, 33], we encountered some difficulty in meeting our target recruitment of 15 participants. Systematic reviews suggest that the majority of clinical trials do not meet their recruitment goals [54, 55] with nearly 20% of trials ending prematurely due to unsuccessful recruitment [56]. Adolescents often have to balance school and after school activities with medical treatment, as well as rely on parents or caregivers for transportation to appointments. Given these constraints, families might have been wary to allow their adolescent to participate in an intervention extending beyond usual care.

We believe developing more realistic eligibility criteria per the Clinical Trials Transformation Initiative [57] might have enhanced our ability to recruit patients. In particular, one of our exclusionary criteria was concurrent mental health treatment. We learned that the majority of adolescent patients were also working with a psychologist or counselor (and indeed, these patients were often the most interested in our study), but physicians could not refer them to our pilot study. We originally decided to exclude these adolescents from the pilot study because some of the content that was practiced during the intervention (e.g., stress reduction, emotional regulation skills) overlaps with techniques and practices that frequently occur in counseling. Therefore, to limit external influence on the effectiveness of the intervention, we included concurrent mental health treatment as an exclusionary criterion. Although maintaining this as an exclusionary criterion in a larger RCT would uphold internal validity, inclusion of adolescents in current mental health treatment would enhance the feasibility of recruitment, along with enhancing the generalizability of the findings to the average adolescent seeking obesity treatment. Other strategies to enhance recruitment might include conducting outreach beyond clinics in order to maximize the number of potential participants, including recruitment within schools or primary care providers’ offices.

Other feasibility outcomes were more promising. Our attendance rate was 85% and consistent with prior mindfulness interventions with adolescents [31, 58]. Anecdotally, participants rarely kept their originally scheduled appointment times, and interventionists had to remain flexible to reschedule participants (often at the last minute) for another appointment time each week. Notably, the participants in our study were given the option between in-person and telemedicine sessions, and the majority chose the latter, suggesting that future work in this area should offer telemedicine delivery of mindfulness sessions to counteract scheduling and transportation barriers. Regarding satisfaction, adolescent feedback was generally quite positive. Adolescents evaluated the program, staff, and benefits of participation highly, perhaps because our intervention included aspects of mindfulness that have been identified as most preferred by adolescents, including hands-on exercises, tools to manage stress in the moment, and techniques to improve the quality of interpersonal relationships [59–61]. Notably, in this pilot we had no method of examining whether the benefits observed post-intervention might have related to participants’ positive relationship with the facilitator, as opposed to the intervention itself, and no way to control for effects between facilitators. A larger efficacy trial should include an attention-matched control group and appropriate modeling of facilitator effects [62].

Participants reported having the most difficulty completing homework and engaging in mindfulness activities on a daily basis due to competing responsibilities like school work or part-time jobs. Prior research with adolescents indicates that a lack of incentives and/or consequences for homework completion reduces motivation to complete it, particularly when it is perceived as competing with their schoolwork [58]. Future programming might consider briefer mindfulness exercises adolescents can take part in anywhere (e.g., three mindful breaths) as opposed to more formal time-consuming practices (e.g., body scan recordings). Programs might also consider the incorporation of mindfulness apps (e.g., Calm, Insight Timer) to facilitate daily practice between appointments and to allow for objective tracking of time spent engaging in mindfulness. Overall, we believe these feasibility outcomes support progression to a larger-scale efficacy trial, pending adaptations to exclusion criteria, recruitment methods, and intervention delivery described herein.

Encouraging trends were observed in some of the health outcomes of interest, although causality cannot be assumed given the lack of a control group. While not statistically significant, BMI was reduced on average by nearly three points, a promising outcome for reducing youth’s risk for chronic disease [63, 64]. This might have been related to youth’s reports that mindfulness increased their ability to monitor their eating habits and pay attention to cues for hunger and fullness. Indeed, youth’s reduction in overeating was approaching significance as they reported nearly two fewer overeating episodes per month. A previous mindfulness intervention with adolescents [33] and several mindfulness interventions with adults [65, 66] have yielded reductions in BMI, although the majority of studies suggest that mindfulness alone is not sufficient to lead to weight loss [17]. In contrast to mindfulness intervention research with adults [67, 68], we did not observe promising reductions in blood pressure as hypothesized. This could have been due to the brief timeframe of the intervention, variety in the time of day during which blood pressure was measured, or the inability to control for medications known to effect blood pressure. All findings must be interpreted with extreme caution given that the confidence intervals of the effect sizes included zero along with the lack of a control group.

Regarding Go/No-Go task performance, we observed a significant difference in reaction time in which participants took a longer time to react on “go” trials following the completion of the intervention. The significant increase in reaction time might be a reflection of increased processing and awareness of the “go” stimuli by participants before responding, suggesting a less impulsive response [69]. No significant changes were observed in the false alarm and omission rates; however, it is worth noting that the false alarm rate—a primary measure of inhibitory control—exhibited a reduction following the intervention with a medium effect size. The lack of significant changes in the false alarm and omission rates might also indicate that the Go/No-Go task is not the most capable measure of inhibitory control. Prior mindfulness research has suggested that changes in neural indicators of response inhibition following a mindfulness intervention have not corresponded with improvements in behavioral performance on the Go/No-Go task [70].

Considering our intervention in the context of broader adolescent obesity treatment recommendations, MBIs seem to align well with established expert committee recommendations for pediatric obesity, including aspects of the seminal model of care put forth by Barlow and colleagues [71, 72]. Stage one of this model addresses prevention of obesity through healthy lifestyle habits (e.g., a nutritious diet, physical activity). Research shows that mindfulness can modify behavioral habits, such as emotional eating and binge eating [17]. Reduction in these habits can help prevent the progression of weight gain in patients at-risk for obesity. Stage two of this model includes providing more support and structure for the adolescent with the goal of achieving healthy eating and physical activity-related behaviors. Guided mindfulness practice provides a structured, goal-oriented intervention for adolescents. MBIs also seem to align with the third tier of this model, which emphasizes a comprehensive multidisciplinary approach with an emphasis on behavioral modification through the use of frequent office visits and specialists. Scheduled mindfulness sessions provide consistency as well as opportunities for clinicians to follow-up on the patient’s care management. Weekly mindfulness sessions require patients to make more frequent visits to the office as well as expand the scope of their treatment. This was demonstrated directly through our pilot study as patients scheduled mindfulness sessions either right after their visits with their physician or on separate occasions.

Limitations of our study include the lack of a control group, which precludes our ability to determine whether any improvements observed in health outcomes were the result of the mindfulness intervention as opposed to outside variables including usual care. Further, the small sample size limited our statistical power to identify significant changes when they occurred and any preliminary results must be interpreted with extreme caution. Finally, our sample was primarily African American and female, with limited representation of other racial/ethnic groups and boys; therefore, the findings may not be generalizable to other samples of adolescents. While limitations exist, ideally, examining the efficacy of this intervention in a larger and more rigorous trial (pending adaptations to recruitment methods and exclusion criteria) will improve outcomes for adolescents with obesity and provide better understanding of how mindfulness might influence eating behaviors.

## Conclusions

In summary, this study aimed to examine whether a brief adjunctive mindfulness intervention was feasible to conduct with adolescent patients with obesity in a pediatric weight management clinic. While participant attendance and satisfaction rates were promising, recruitment and retention proved more challenging. The lessons learned will inform a larger efficacy trial involving more extensive examination of CVD functioning and including an attention-matched control group. Should this intervention prove successful, it could be easily translated into other weight management clinics, given that the intervention is manualized and does not need to be implemented by clinicians with extensive psychological training. Mindfulness may be a successful tool for improving emotional regulation and decision-making in adolescents with obesity, leading to improved weight loss, health outcomes, and quality of life.

## Supplementary information

**Additional file 1:** Responses to open-ended satisfaction questions for all program completers

## Data Availability

The datasets used and/or analyzed during the current study are available from the corresponding author on reasonable request.
