# Supplementary material for: A pilot open-label feasibility trial examining an adjunctive mindfulness intervention for adolescents with obesity
Source: Pilot Feasibility Stud. 2020 Jun 6;6:79. doi: 10.1186/s40814-020-00621-1 (PMC7275381; doi:10.1186/s40814-020-00621-1)
Supplement: Supplementary file 1 — Additional file 1: Responses to open-ended satisfaction questions for all program completers [file 40814_2020_621_MOESM1_ESM.docx]

Appendix I.

*Responses to Open-Ended Satisfaction Questions for all Program Completers (n = 8)*

| Satisfactory Questions | Participant Responses |
| --- | --- |
| What were the benefits of participating in this study? | \| *Help[ed] manage weight loss and emotions* \| \| --- \| \| *[Exposure to] Mindfulness and health things* \| \| *Help[ed] enjoy food more and in smaller quantities* \| \| *Learning more about mindfulness and sharing it with others* \| \| *Losing weight and watching what I eat* \| \| *Learning when I'm actually hungry and when I'm stress eating* \| \| *Learning ways to improve and money* \| \| *Helped control eating and portions* \| |
| What were the challenges of participating in this study? | \| *Find good time for [telemedicine] calls* \| \| --- \| \| *Coming every Tuesday* \| \| *Freeing up time each week* \| \| *Call connection [for telemedicine session]* \| \| *Watching what I eat* \| \| *Wishing good to people who have been bad to me* \| \| *Remembering homework* \| \| *Staying awake* \| |
| What barriers, if any, did you encounter to participate in mindfulness? | \| *Connection to [telemedicine] calls* \| \| --- \| \| *My health* \| \| *None* \| \| *Forgetting about mindfulness when it is needed the most* \| \| *Eating* \| \| *Impatient with the 10-minute body scan* \| \| *None* \| \| *None* \| |
| What changes have you noticed in yourself after participating in this study? | \| *Dealing with my emotions* \| \| --- \| \| *My health* \| \| *Mindfulness and meditation in daily routine* \| \| *I've become more peaceful and able to control my emotion* \| \| *Less eating* \| \| *Being able to calm down easier* \| \| *Thinking about strategies before unhealthy choices* \| \| *Breathing more and falling asleep easier* \| |
| What improvements would you suggest for future programs like this? | \| *None* \| \| --- \| \| *More about health* \| \| *None* \| \| *Better way to communicate with better quality and more fun activities* \| \| *None* \| \| *None* \| \| *None* \| \| *More with breathing and standing* \| |
